# Letter from Dr. Holcomb

**Published:** 1860-04

**Authors:** 


— Dr. Wm. F. Holcomb, to whose kindness we are indebted for the paper by Prof. Langenbeck, informs us that the translation was revised by Prof. Langenbeck himself.

From the letter which accompanied the paper we select a few paragraphs, as they afford important information relative to the various schools of Europe, judged by one who has at two separate visits passed several years among them.

Dr. Holcomb writes us, "that if a young man calculates to reap much advantage by coming to Europe and staying here and there, 'on the wing,' and returning home in a year or eighteen months, as so many do, he greatly misapplies his time and money. An American doctor—just '*fledged*'—had better spend a year in the hospitals of any of our large cities (if he has not done so) before coming to Europe; and unless he can speak or understand perfectly French and German, or can stay two or three years in Europe, he had better not waste his time or money by going to Paris or Germany. I have found Dublin to be he best place for American doctors who come abroad to stay a year or so, though its advantages are seized by a few only. The Dublin Medical and Surgical Professors are the best educated, accomplished, noble gentlemen I have yet seen anywhere, and the advantages for hospital study in Dublin are almost unlimited.

"I think the advantages in Germany for the study of Pathology, Physiology, and Microscopical Anatomy are far superior to those in France, but a man must make up his mind to go *slowly*—as the German always does—but, nevertheless, most *surely*, towards a thorough knowledge of what he studies. Berlin, Vienna, Würtzburg, Erlangen, each have their advantages. Graefe, of Berlin; Donders, of Utrecht; Arlt, of Vienna, are the great teachers of ocular diseases. Virchow is the great pathologist of the day, though he is still a young man—under 40. He certainly has very great power in grasping facts, and distinguishing quickly political, religious, or physiological truths or fallacies. He is as great a Republican as he is a Physiologist. He has promised me a photograph of the Pathological Institute here, and with it I will send you a description of the same, and the plan of study here. It is most perfect, and one *must* learn if he wishes to."

